# More bipolar than bipolar disorder – a polygenic risk score analysis of postpartum psychosis

**DOI:** 10.1192/j.eurpsy.2023.1079

**Published:** 2023-07-19

**Authors:** E. A. Hörbeck, L. Jonsson, E. Pålsson, M. Landén

**Affiliations:** 1 Department of Psychiatry and Neurochemistry, Institute of Neuroscience and physiology, Sahlgrenska Academy, University of Gothenburg; 2 Psychosis Clinic, Sahlgrenska University Hospital, Gothenburg, Sweden

## Abstract

**Introduction:**

Postpartum psychosis is a rare psychiatric emergency, occurring days to weeks after 1-2 per 1000 deliveries. Its low prevalence makes it difficult to recruit enough participants to investigate the underlying pathophysiology. It is epidemiologically linked to bipolar disorder, which one study also found it to resemble in genetic susceptibility for psychiatric disorders (Di Florio *et al*. Lancet Psych 2021; 8: 1045–52).

**Objectives:**

In this study we aim to investigate polygenic liability for psychiatric disorders in two new Swedish postpartum psychosis cohorts.

**Methods:**

Cases with postpartum psychosis, defined as a psychiatric hospitalization within 6 weeks after delivery, and/or receiving a diagnosis of F53.1 (ICD 10) or 294.40 (ICD 8.), parous women with severe mental illness without postpartum psychosis, and healthy parous controls were identified in two Swedish genetic studies: the Swedish bipolar collection (SWEBIC) and Predictors for ECT (PREFECT). Polygenic risk scores (PRS) were calculated from summary statistics from genome wide studies on bipolar disorder (Mullins *et al.* Nat Genet 2021; 53 817-829), schizophrenia (Trubetskoy *et al.* Nature 2022; 604 502-508) and major depression (Wray *et al*. Nat Genet. 2018; 50 668-681). The p-value thresholds best predicting their respective phenotype were used in logistic regression analyses with the first six principal components and genotyping platform as confounders.

**Results:**

We identified 176 patients with postpartum psychosis and genetic information (N(SWEBIC)=126, N(PREFECT)=50). Compared with healthy parous women, patients with postpartum psychosis had significantly higher PRS for bipolar disorder (SWEBIC: odds ratio [OR] 2.6 (95% confidence interval [CI] 1.9-3.5), PREFECT: OR 2.4 (95% CI 1.8-3.2), Figure 1.) and schizophrenia (SWEBIC: OR 1.6 (95% CI 1.2-2.2), PREFECT: OR 1.8 (95%; CI 1.3-2.5)). Patients with postpartum psychosis had significantly higher PRS for bipolar disorder (SWEBIC: OR 1.4 (95% CI 1.2-1.8), PREFECT: OR 1.5 (95% CI 1.1-2)) compared with parous women with severe mental illness without postpartum psychosis. We found no associations with major depression PRS in either cohort.

**Image:**

**Figure:**
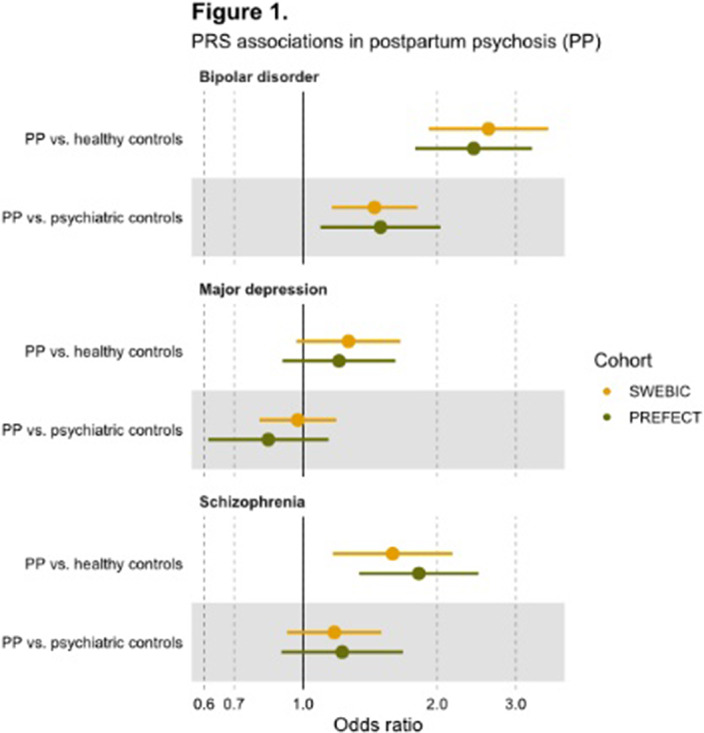

**Conclusions:**

We replicated previous findings of significantly higher PRS for bipolar disorder and schizophrenia in postpartum psychosis compared with healthy controls. In contrast to previous research, we find postpartum psychosis cases to have higher PRS for bipolar disorder than bipolar disorder cases. Our findings highlight the genetic influence in postpartum psychosis and support previous genetic and epidemiological evidence that postpartum psychosis lies on the bipolar spectrum.

**Disclosure of Interest:**

None Declared

